# Sensorimotor adaptation impedes perturbation detection in grasping

**DOI:** 10.3758/s13423-024-02543-y

**Published:** 2024-07-24

**Authors:** Carl Müller, Alexandra Bendixen, Karl Kopiske

**Affiliations:** Cognitive Systems Lab, Institute of Physics, Chemnitz University of Technology, 09126 Chemnitz, Germany

**Keywords:** Visual perception, Haptic perception, Perception and action, Sensorimotor adaptation, Just-noticeable difference (JND)

## Abstract

Humans achieve skilled actions by continuously correcting for motor errors or perceptual misjudgments, a process called *sensorimotor adaptation*. This can occur with the actor both detecting (explicitly) and not detecting the error (implicitly). We investigated how the magnitude of a perturbation and the corresponding error signal each contribute to the detection of a size perturbation during interaction with real-world objects. Participants grasped cuboids of different lengths in a mirror-setup allowing us to present different sizes for seen and felt cuboids, respectively. Visuo-haptic size mismatches (perturbations) were introduced either abruptly or followed a sinusoidal schedule. These schedules dissociated the error signal from the visuo-haptic mismatch: Participants could fully adapt their grip and reduce the error when a perturbation was introduced abruptly and then stayed the same, but not with a constantly changing sinusoidal perturbation. We compared participants’ performance in a two-alternative forced choice (2AFC) task where participants judged these mismatches, and modelled error-correction in grasping movements by looking at changes in maximum grip apertures, measured using motion tracking. We found similar mismatch-detection performance with sinusoidal perturbation schedules and the first trial after an abrupt change, but decreasing performance over further trials for the latter. This is consistent with the idea that reduced error signals following adaptation make it harder to detect perturbations. Error-correction parameters indicated stronger error-correction in abruptly introduced perturbations. However, we saw no correlation between error-correction and overall mismatch-detection performance. This emphasizes the distinct contributions of the perturbation magnitude and the error signal in helping participants detect sensory perturbations.

## Introduction

### Sensorimotor adaptation and sensory error

Interacting with our environment is a complex process that involves continuous recalibration of our actions (Helmholtz, 1867; Woodworth, 1899). We observe an object, we reach out to manipulate it, slowly and a bit clumsily at first, but over several manipulations, we become more proficient. This process of learning to perform actions and reducing associated motor error is referred to as *sensorimotor learning* (Krakauer & Mazzoni, 2011). Systematic errors are often of particular interest: Not only can effects of learning on them be very large compared to that on random errors (Bingham & Mon-Williams, 2013; Burge et al., 2008), they can also be experimentally manipulated, and in different sensory modalities, allowing us to disentangle the contributions of the respective sensory channels to specific actions (Ernst & Banks, 2002). Indeed, inducing errors (that is, perturbing actions), for example through mismatches between sensory channels, is a common method to investigate how humans deal with motor errors more generally.

Typically, we consider sensorimotor adaptation to be mainly a consequence of correcting motor errors (Shadmehr et al., 2010). For example, after reaching towards a target and erring to the left, one would respond by moving the arm further to the right the next time (Van Dam & Ernst, 2013); after failing to grasp an unexpectedly large object, one would open the hand more on the next grasp (Säfström & Edin, 2004). Thus, the action would be corrected based on information from the previous grasp to ensure that another such grasp would be successful.

### Detecting sensory errors

During adaptation, humans often notice that some adjustment is needed. Experimentally, the awareness of perturbations may be manipulated by (i) using an explicit instruction (Miyamoto et al., 2020; Taylor & Ivry, 2011), (ii) distracting participants (Mariscal et al., 2020), or (iii) changing some inherent properties of the perturbation – for example, making it very large or introducing it gradually rather than abruptly to mask the mismatch (Kagerer et al., 1997; Orban De Xivry et al., 2013). The third example may be the most ecologically valid, but also the most difficult, as it relies on various sources of information the influence of which is only known indirectly.

Take a large size mismatch in grasping, a participant picking up an object that visually appears smaller than it really is. The participant may detect that their fingers do not touch the object at quite the same time nor with the speed and force that they normally would. Next, they may detect that the felt (*haptic*) size of the object is different from the seen (*visual*) size, and its unexpected weight. Thus, there are many different sensory inputs, each with its own just-noticeable difference (JND; Fechner, 1860), and often not linearly dependent on the mismatch (Jeannerod, 1986). Thus arises our main question: What makes a perturbation detectable?

### Dissociating mismatch and error signal

It is intuitively plausible that the magnitude of a perturbation should matter in terms of how easy it is to detect (Hudson & Landy, 2012; Modchalingam et al., 2019), and experimental results back this up (Gaffin-Cahn et al., 2019). However, differential effects of introducing perturbations abruptly versus gradually (Modchalingam et al., 2023; Orban De Xivry et al., 2013) demonstrate that this is not the whole story: For example, Orban De Xivry et al. (2013) found motor-evoked potentials to change following abruptly but not gradually introduced force-field perturbations, and previous work also found stronger learning for greater EMG (electromyographic)-feedback response to error (Albert & Shadmehr, 2016). In a similar vein, Modchalingam et al. (2023) showed that gradually introduced perturbations can lead to a larger implicit adaptation, and that identifying the schedule of introducing the perturbation matters. One potential explanation for this is that as motor actions change through error-correction, this in turn changes the error signal.

**Fig. 1:**
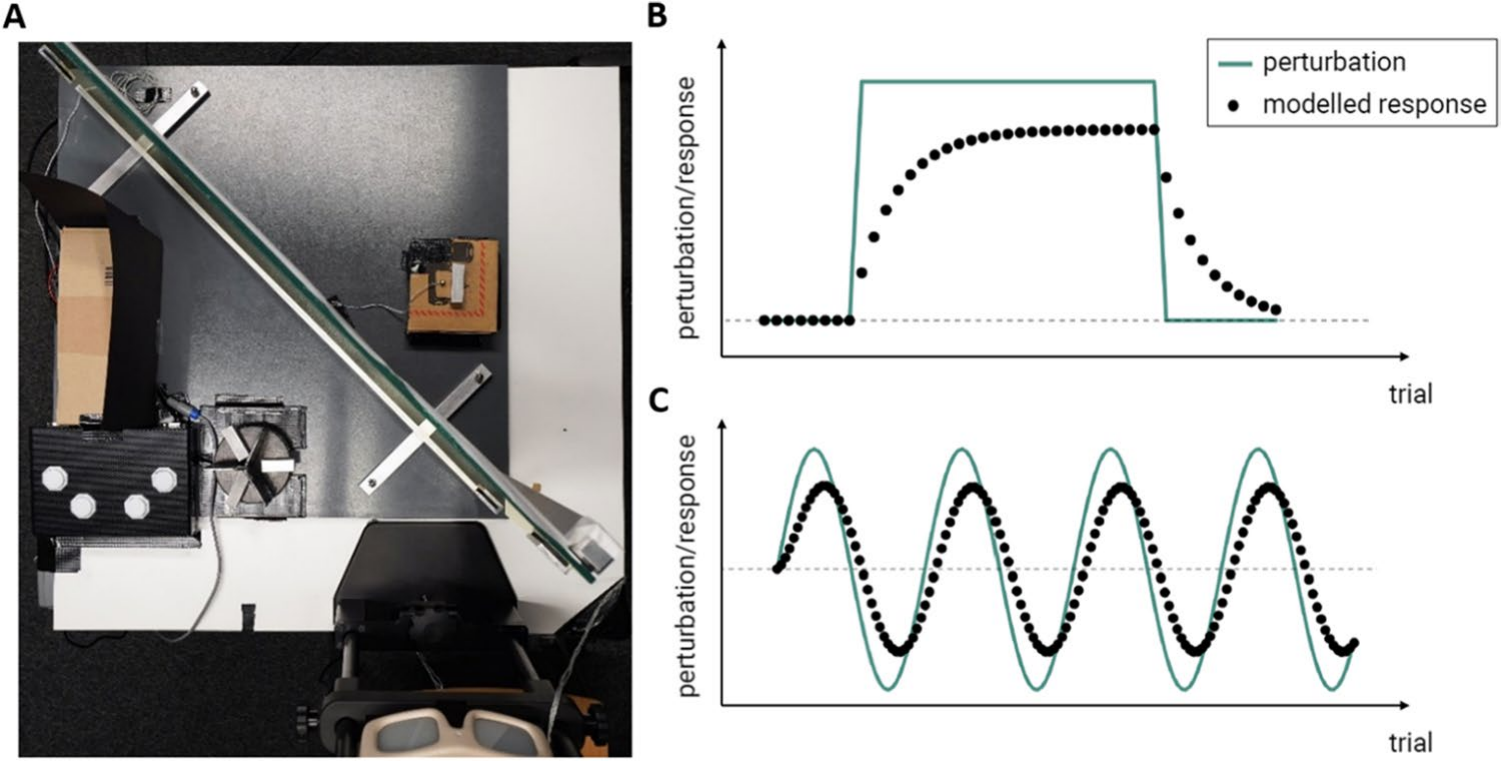
Experimental setup and different perturbation schedules. **Left: A**: Bird's-eye view of the experimental setup. Participants were sitting at a table, wearing the LCD goggles, and looking in a front-silvered mirror. In front of this mirror, the seen objects were placed on the turntable besides the response box for the two-alternative forced choice (2AFC) task. Behind the mirror and not visible to the participant, the felt objects were positioned at the imaginary same position as the seen objects appear when looking in the mirror. **Right**: Schematic illustration of two different perturbation schedules and modelled responses (y axis) across trials (x axis). The green line indicates the perturbation, the black dots show the corresponding responses with adaptation modelled following equation 3 and with parameters $A$ = 0.95 and $b$ = 0.2, similar to those obtained using a similar setup and the same model in Kopiske et al. (2017). Panels are adapted from Hudson and Landy (2012). **B**: Perturbation occurs abruptly after a baseline phase and ends abruptly to return to baseline level for the washout phase. Responses show the typical exponential function towards an asymptote, followed by an exponential decay during washout. **C**: Perturbation is induced gradually following a sinusoidal schedule. The adaptation shows a shift in phase and a slightly reduced magnitude

With an abrupt perturbation schedule (Fig. 1), the change in size difference between seen and felt size (*mismatch*) occurs only in the first perturbed trial but the mismatch itself remains constant over all following trials. This implies initially larger error signals for the first trial – however, through sensorimotor adaptation the error signal decreases with every trial, which might affect detectability of the mismatch. For sinusoidal perturbations on the other hand, the size difference changes more subtly with every trial. Trial-by-trial error correction models will then predict the error signal to be smaller initially but without systematic decline, and no asymptotic behavior. If participants anticipated the sinusoidal perturbation schedule, asymptotically decreasing error would also be possible here – yet empirically, adaptation under noisy conditions has been shown to be much more non-specific (Wei et al., 2010). Knowing how the detection of perturbation evolves over time for different perturbation schedules could therefore be an important component of the more general question of what inherent properties make perturbations detectable.

Our experiments investigated the respective contributions of these two factors by dissociating the *mismatch* (magnitude of the perturbation) and the *sensory error signal* (i.e., the difference between the expected outcome and the observed outcome) and assessing their impact on perturbation detection and adaptation in a grasping task. To do this, we asked participants to grasp real cuboids of different lengths in a mirror-setup with visuo-haptic mismatches and then compare felt and seen lengths. Using either an abrupt or a sinusoidal (Hudson & Landy, 2012) perturbation schedule to introduce size mismatches allowed us to dissociate the mismatch and the error signal. If the error signal is crucial for detecting perturbations, we would expect a high detection performance for the first perturbed trials in abrupt schedules followed by decreasing performance over the subsequent trials with the constant mismatch, but no decreasing detection performance for sinusoidal perturbations with a consistently moderate error signal.

## Methods

We asked participants to grasp cuboids while looking in a front-silvered mirror (Fig. 1A), allowing us to present different sizes for seen objects (in front of the mirror) and the felt object (behind the mirror), respectively. As a perturbation schedule that would allow participants to fully adapt, we used an abrupt schedule (Fig. 1B) consisting of a short baseline period followed by a constant mismatch between seen and felt size. To dissociate the magnitude of the mismatch from the error signal (difference between perturbation (green line) and the modelled response (black dots)), we also introduced mismatches more gradually on a trial-wise base following a sinusoidal schedule (Fig. 1C) (suggested, e.g., by Hudson & Landy, 2012), resulting in initially smaller error signals without systematic decrease. The mismatch magnitudes from the abrupt schedules were used as the maximum mismatch of the different sinusoids. Participants were then asked to judge the relative size of the objects to assess perturbation detection. While this two-alternative forced choice (2AFC) question did not allow us to infer detectability on a trial-wise basis, since there is no way to distinguish between a correct guess by chance and the participant knowing the correct answer, we could analyze mean performance changes over trials, taking chance level into account.

We first conducted a pilot experiment (N = 24) to test whether there would be any difference in detection performance between the two perturbation schedules. Analyses showed a decrease in detection performance over trials when perturbations were introduced abruptly and then stayed constant, compared to a relatively constant performance over time (as well as overall better performance) with sinusoidal perturbations. We then aimed to replicate this in our main experiment with a larger sample (N = 48) as well as improved methods.

### Participants

Participants were recruited via a TU-Chemnitz online mailing list. All were right-handed by self-report, had no motor impairments in their arm, and had normal or corrected-to-normal vision. All participants reported being sufficiently rested and focused in a questionnaire administered prior to the experiment, were naïve to the hypotheses, and were debriefed after the experiment.

Our pilot experiment was conducted with a total of N = 24 participants, of which 23 were analyzed (one excluded due to a high proportion of missing data), including 16 women and seven men with an average age of 23.5 years (between 19 and 32 years). This sample size gave us sufficient statistical power to detect a medium to large effect (power of .8 for d = 0.6; Cohen, 1988); however, we did not have a reasonable estimate for the expected effect in the size-comparison task before the pilot experiment. In the main experiment we analyzed a sample of *N* = 48 participants including 34 women and 14 men with an average age of 23.1 years (between 18 and 53 years). As the difference in JNDs in our pilot experiment did indeed turn out to be a medium effect (d = 0.6), we based our power analysis on a medium effect of d = 0.5, for which we needed N = 44 to achieve .9 power. Both experiments lasted about 2 h and participants received either course credit or a monetary reimbursement of 8 €/h in the pilot experiment or 10 €/h in the main experiment. All experimental procedures were in accordance with the 2013 Declaration of Helsinki and were approved by the appropriate body (pilot: Chemnitz University of Technology, Faculty of Behavioral and Social Sciences ethics committee, reference no. V-329-PHKP-WET-Adaptation-10052019; main experiment: Chemnitz University of Technology ethics committee, reference no. 101568507). Participants had been fully informed about the study prior the experiment, and participant data were protected according to institutional regulations.

### Setup and procedure

Participants were seated at a table, 30 cm in front of a front-silvered mirror aligned 45° to their gaze orientation (Fig. 1A), their head in a chin rest. They saw aluminum cuboids with a 15 mm × 15 mm base (seen objects), while behind the mirror at the same position where the seen objects appeared to be, cuboids – whose length was sometimes perturbed – for grasping (felt objects) were placed. Participants could not look behind the mirror and thus did not see the felt objects or their own hand during grasping. The grasping movement was tracked (for 5 s at 200 Hz in the pilot and for 3 s at 500 Hz in the main experiment, respectively) using the Optotrak 3D Investigator (Northern Digital Inc., Waterloo, Canada) with four active markers fixated on the thumb, index finger, the wrist, and near the felt object to enable us to estimate the hand’s distance to the target.

In the pilot experiment, the seen objects were constant over blocks (but varied between blocks at either 40 mm or 45 mm length), whereas the felt objects were replaced with every trial by the experimenter (or inconspicuously repositioned if the felt object remained the same). Each trial started with a verbal signal by the experimenter (“jetzt,” German for “now”). Participants had their right hand in a starting position on the table and were instructed to directly grasp the object behind the mirror with their thumb and index finger in a precision grip (Napier, 1956) and lift it up at about 5 cm. They then verbally indicated whether the felt object was larger or smaller than the seen object.

In the main experiment, we slightly modified the setup and procedure. The setup was improved by using more seen objects with different lengths (40 mm, 44 mm, and 48 mm) varying between trials by using a rotation disk. To control visibility and to indicate the start of a trial, we used LCD shutter goggles (PLATO goggles; Milgram, 1987). At the start of each trial, the LCD goggles opened, and participants again saw the cuboid in the mirror, grasped it, and indicated whether the felt object was larger or smaller than the seen object, this time using a response box. We emphasized accuracy and not speed in both experiments. After the response, the LCD goggles closed, and the seen object changed while the experimenter prepared the corresponding felt object.

### Stimuli and manipulations

The pilot and the main experiment consisted of one practice block (12 trials) at the beginning and 12 experimental blocks, separated in six blocks following different perturbation schedules. These schedules varied between *abrupt* (24 trials, four non-perturbed baseline trials at the beginning and the end of a block, respectively) and *sinusoidal* (36 trials, three cycles; first trial of each block was non-perturbed). In the pilot experiment, all cycles started with a positive perturbation; in the main experiment, half of the sinusoidal blocks started with a negative perturbation. The lengths of the seen cuboids were either 40 mm or 45 mm in the pilot experiment, with a block-wise change, and 40 mm, 44 mm, or 48 mm with a randomized trial-wise change in the main experiment. The corresponding felt cuboids for abrupt and maximum mismatch of the sinusoid were presented in the pilot experiment with perturbation magnitudes of ± 4 mm, ± 8mm, and ± 12 mm, and in the main experiment with ± 3 mm, ± 6 mm, and ± 12 mm relatively to these seen object sizes. These felt cuboids varied between 28 mm and 60 mm with a minimal step size of 0.5 mm for the sinusoidal schedule. For the pilot experiment, the smallest perturbation magnitude was chosen to be roughly the size of the JNDs for visual-haptic size comparisons as reported in Hillis et al. (2002). The magnitude for the main experiment was adapted from the JNDs of the pilot experiment with a larger range to capture possible smaller JNDs. Block order for the pilot experiment was fully randomized and for the main experiment counterbalanced over participants using a combination of four 12 x 12 Latin squares.

### Data processing

For interpolating the motion capture data from the Optotrak measurements to deal with missing values, we applied a cubic-spline and used a Savitzky–Golay Filter (Savitzky & Golay, 1964) with a window of 200 ms to smooth the signal. These data were analyzed in R (R Core Team, 2022), extracting the maximum grip aperture (MGA), movement time (time difference between movement start and touching the object), and time to MGA (time difference relative to movement start). We set the start of the grasping movement through a velocity criterion (thumb and index velocity > 0.05 m/s), and we used a combination of an aperture-velocity criterion and a position criterion to determine when the object was “touched” (aperture velocity < 0.1 m/s (pilot) or < 0.075 m/s (main experiment), and mean point between index finger and thumb nearer than 300 mm (pilot) or 150 mm (main experiment) to the center of the target object; these differences resulted from using two slightly different sets of markers in the two experiments). Such a combination of criteria has been shown to be robust in grasp-movement segmentation (Schot et al., 2010). The MGA then was defined as the maximum aperture before the “touched” event, extracted for each trial and our foundation for further adaptation models. Trials were excluded from analysis if (i) the MGA was at a point where the trajectory had been interpolated, (ii) more than 20% of frames between movement start and touching the object were missing, (iii) the detected MGA was implausibly small (i.e., smaller than the object length), or (iv) the MGA was detected as an outlier for being more than 3 interquartile ranges removed from the participant's median MGA for the same seen and felt size. This way, we excluded 5.3 % (pilot) and 1.9 % (main) of trials from analysis.

### Modelling grasping and error-correction

When grasping, people have to adjust their grip aperture to the size of the different objects to be grasped. How they grasp different objects has been calibrated through thousands of previous grasps to ensure successful and comfortable grasps, and is often quantified in terms of the MGA. This measure has the desirable property that it scales reliably and monotonically with object size (Smeets & Brenner, 1999). However, people usually open their fingers more widely than the actual object size, and do not scale their grip perfectly with object size, so the object size has to be related to the typical MGA via a response function. This is typically modelled as a linear function consisting of an intercept $int$ and a $slope$ (Säfström & Edin, 2004) that determines scaling with seen object size ${v}_{t}$:

1 $${MGA}_{{v}_{t}}=int+slope*{v}_{t}$$

This formula describes non-perturbed everyday grasping with identical seen and felt object size. We can then model the participants’ response to perturbations by introducing a state ${x}_{t}$ representing a visuomotor mapping (Hayashi et al., 2016) that can be thought of as an alteration to movement planning when the participant sees the object and prepares to grasp it, which in the model is simply added to seen object size ${v}_{t}$:

2 $${MGA}_{{mod}_{t}}=int+slope*\left({v}_{t}+{x}_{t}\right)$$

For a normal non-perturbed grasp, ${x}_{t}=0$ and the response function is identical to equation 1, as no adjustment to a perturbation has taken place. When introducing size perturbations in which seen size ${v}_{t}$ and felt size ${h}_{t}$ are dissociated, the adjustment can be modelled using a linear state-space model (Wolpert et al., 1995) in which ${x}_{t}$ is updated from trial to trial. Such models are commonly used to describe visuomotor adaptation (Thoroughman & Shadmehr, 2000), formalizing the idea of sensorimotor adaptation to be a consequence of correcting motor errors on a trial-wise basis, and are frequently written as:

3 $${x}_{t+1}=A{x}_{t}-b{E}_{t}$$

where ${x}_{t}$ is the state at time point $t$, changing from trial to trial depending on the error term. $A$ and $b$ are parameters representing state retention and error-correction, bounded between 0 and 1, respectively. An $A=0$ means no retention of the previous state, whereas $A=1$ indicates perfect retention. A value of $b=0$ means no error-correction from one trial to the next, while $b=1$ indicates complete error-correction. The error signal ${E}_{t}$ reflects the amount by which the participant’s grip was too large or too small, and so leads to an adjustment of the expected object size for the next grasp. As the error signal only depends on the haptic feedback, we use the felt size ${h}_{t}$ and the response function from equation 1 to estimate which MGA would result in a comfortable grip given the object being grasped, and compare this to the observed (measured) MGA. Thus, the calculation rests on the difference between the current observed ${MGA}_{t}$ and the MGA based on the felt size:

4 $$\begin{array}{l}{E}_{t}={MGA}_{{h}_{t}}- {MGA}_{t}\\ =\left(int+slope*{h}_{t}\right)- {MGA}_{t}\end{array}$$

Further, calculating the next state based on the error signal, error-correction parameter $b$ and retention parameter $A$ were fitted as free parameters on a block-wise basis. As we had previously done (Kopiske et al., 2017), we decided to also fit the intercept, but not the slope of the response function. This was for two main reasons: One, given its large variability and absolute numeric values, a poorly estimated intercept would mask any other effects in the data. Two, the slope parameter is inherently related to others, such as $b$, as both indicate a responsiveness (to size, or to errors); thus, we did not fit this parameter and used the overall mean slope. Free parameters were fitted using the nloptr package in R (Ypma, 2014) to minimize the root mean squared error (RMSE) between the observed MGAs and the modelled MGAs given state ${x}_{t}$ and the seen sizes, described in equation 5 for a block of $n$ trials:

5 $$RMSE=\sqrt{\frac{{\sum }_{t=1}^{n}{({MGA}_{{mod}_{t}}-{MGA}_{t})}^{2}}{n}}$$

Comparing the observed ${MGA}_{t}$ to the modelled ${MGA}_{{mod}_{t}}$ given seen size and the adjusted state follows the intuition that vision is used for action planning (as it is available before the action) and that the visuomotor mapping that is updated via the state ${x}_{t}$ relates seen object size to the associated grasp. Haptics on the other hand in our model affect grip apertures indirectly by updating the visuomotor mapping (more formally, the state) through error-correction.^1^

### Main analyses

Adaptation parameters from the MGA modelling were submitted to repeated-measures analyses of variance (rmANOVAs) with factors *perturbation schedule* and *perturbation magnitude* to test if the perturbation schedule or magnitude affected the extent of adaptation. For assessing perturbation detection performance and trends over trials, we fitted participant-wise linear slopes over the percentage correct across trials per *perturbation schedule* (abrupt, sinusoidal) and *perturbation magnitude* (pilot: 4mm, 8mm, 12mm; main: 3mm, 6mm, 12mm) and conducted a 2 x 3 rmANOVA with these two factors and the slopes as the dependent variable. Trials in which seen and felt objects were equally large were excluded from these analyses, because the correct answer (equal) was not available for the participants. Additionally, we calculated separate rmANOVAs on absolute percent correct, testing the main effect of *perturbation magnitude* for each schedule. We then computed JNDs for each participant and perturbation schedule by fitting a cumulative normal distribution psychometric function using the quickpsy package (Linares & López-Moliner, 2016) in R and compared them using paired t-tests. JNDs integrate the information of all trials while taking perturbation magnitude into account, providing an overview of detection performance and a straightforward way to compare performance in the two schedules. Further, we looked at the correlation of detection slopes and JNDs per participant with the mean error-correction parameter for each participant, each averaged across perturbation schedules, to investigate inter-individual effects of detection performance and adaptation. Given the importance of null differences, we also calculated Bayes factors for differences in the mean error-correction parameters between schedules, for differences in the overall mean JNDs (Rouder et al., 2009), each using a medium-width prior (r = 0.707 as used by Morey & Rouder, 2018), as well as for all correlation analyses (with a medium-width prior of r = 0.333). We report Cohen’s d (Cohen, 1988) as an effect size. Data and analysis scripts are available via the Open Science Framework at: https://osf.io/2569y/.

## Results

### Adaptive behavior and error-correction

**Fig. 2:**
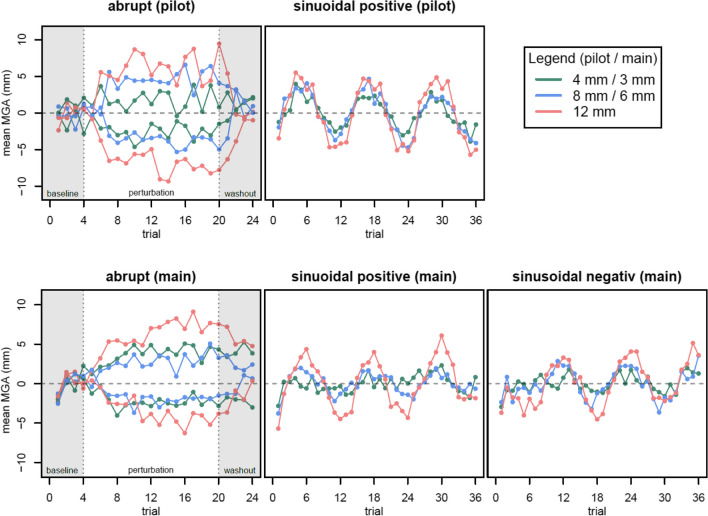
Mean maximum grip apertures (MGAs) per trial (baseline-corrected) over all participants, separated by perturbation schedule for all perturbation magnitudes (**upper panel:** pilot, **bottom panel:** main). Non-perturbed trials for the abrupt schedule are shown transparently. MGAs roughly show the abrupt perturbation pattern (increasing or decreasing correspondingly) and show a phasic pattern in the sinusoidal perturbation blocks. Data from the main experiment are moreover divided into positive and negative sinusoidal perturbation, following their perturbation magnitude at the block start, resulting in an anti-phasic pattern of the mean MGAs

First we investigated whether participants indeed adapted their grips to perturbations. We analyzed if there were effects of the *perturbation schedule* and *magnitude* on the adaptation of grasping by looking at the MGA (Fig. 2). We tested the scaling of object sizes with the MGAs by fitting a response function (*MGA ~ seen size*) over the mean MGAs per participant over all seen object sizes. We found mean slopes of 0.93 (pilot) and 0.32 (main experiment), so MGAs scaled with the object sizes, albeit somewhat weakly in the more complex main experiment (Smeets & Brenner, 1999).

**Fig. 3:**
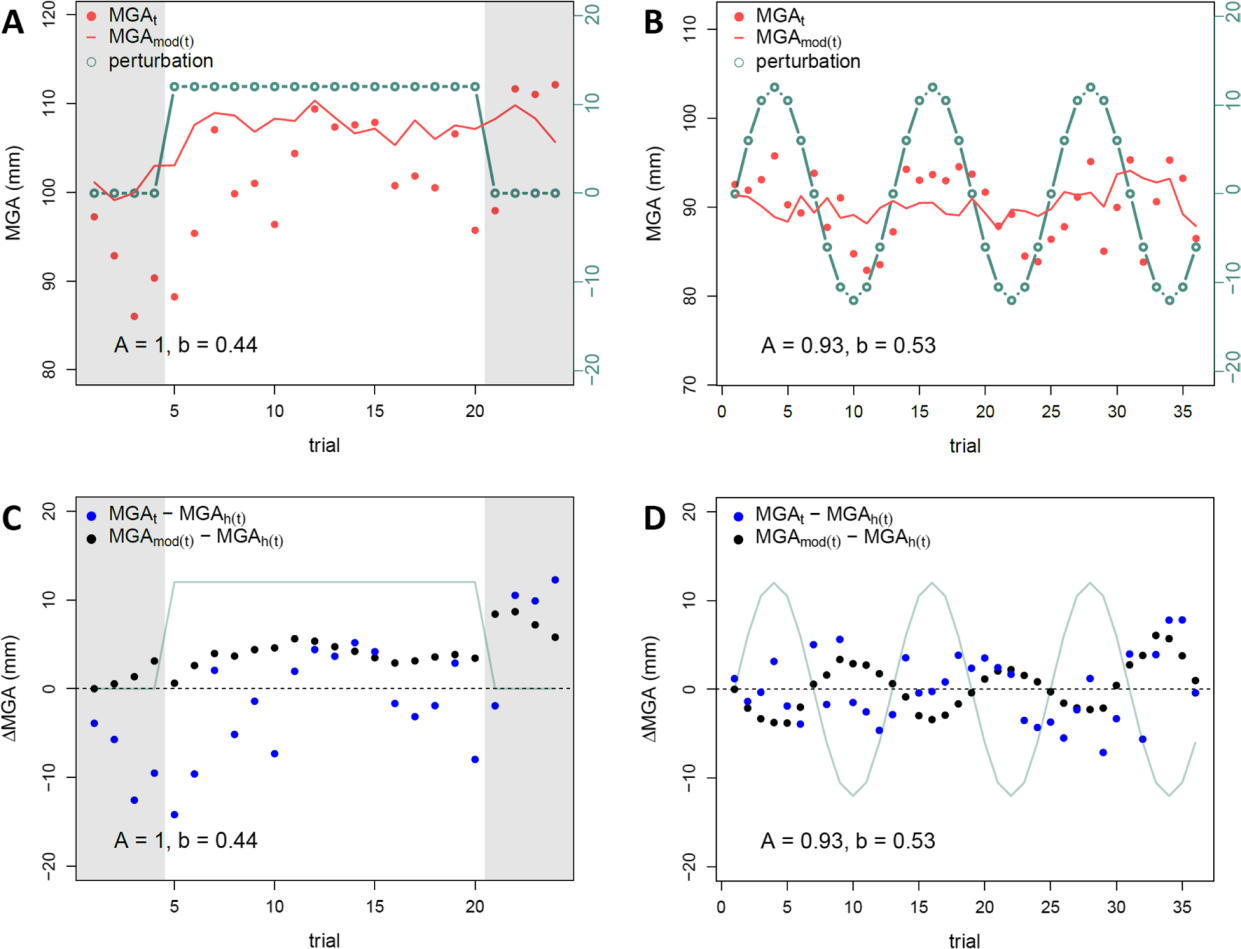
Example of modelled maximum grip apertures (MGAs) with adaptation parameters. Error-correction model (red line) applied at the observed MGAs (red dots indicate raw data) for the abrupt (**A**) and sinusoidal (**B**) perturbation schedule (green dot-line) of one participant of the main experiment. Panels **C** and **D** show the differences between the observed ${MGA}_{t}$ (blue dots) and the modelled ${MGA}_{{mod}_{t}}$ given seen size (black dots) and the expected ${MGA}_{{h}_{t}}$ given the felt size for the corresponding block to panels **A** and **B**, respectively. Thin green lines show the perturbation. Panels **A** and **B** show how the model ${MGA}_{{mod}_{t}}$ fits the observed ${MGA}_{t}$. Panels **C** and **D** show how model predictions are updated: Whenever ${MGA}_{t}$ - ${MGA}_{{h}_{t}}$ is positive, ${MGA}_{{mod}_{t}}$ is corrected downwards (because the previous grasp was “too large”), by an amount scaled by $b$ and the slope of the response function and vice versa. An error-correction parameter $b$ (e.g., of 0.44 for the block plotted in panels **A** and **C**) indicates a mean correction of the deviation from the real MGA to the predicted state of 44 % in each trial. Note the model (noisily) approaching an asymptote in the abrupt perturbation schedule (**A** and **C**) and lagging behind the perturbation in the sinusoidal perturbation schedule (**C** and **D**)

To assess the extent of sensorimotor adaptation, we applied the error-correction model (equation 3) to the observed MGA for each block of each participant (an example is shown in Fig. 3, with mean parameters in Table 1). We found significant differences for the error-correction parameter ($b$) in the pilot experiment depending on the *perturbation schedule*, *F*(1,22) = 11.94, *p *= .002, but not the *perturbation magnitude*, *F*(2,44) = 1.84, *p *= .171, with no interaction*, F*(2,44) = 2.91, *p *= .065. A Bayesian t-test comparing the two schedules showed the same effect (BF_10_ = 17.5), confirming that mean error-correction parameters $b$ were larger for abrupt perturbations (0.28) than for sinusoidal perturbations (0.20). These differences were replicated in the main experiment, with a main effect for *perturbation schedule*, *F*(1,47) = 148.19, *p *< .001, and $b$*`*s of 0.45 for abrupt and 0.20 for sinusoidal, respectively (BF_10_ > 1000), and again with no effect for *perturbation magnitude*, *F*(5,235) = 0.85, *p *= .516, but with a significant interaction, *F*(5,235) = 3.21, *p* = .008, indicating that $b$*`*s were not entirely independent of *magnitude* (Table 1).

**Table 1:** Mean adaptation parameters with 95% confidence intervals

Pilot / Main	magnitude	-12 mm	-8 mm / -6 mm	-4 mm / -3 mm	4 mm / 3 mm	8 mm / 6 mm	12 mm
	schedule						
Pilot experiment	Abrupt	A = 0.81 [0.72;0.89] b = 0.27 [0.19;0.34]	A = 0.82 [0.73;0.90] b = 0.24 [0.18;0.31]	A = 0.77 [0.69;0.84] b = 0.32 [0.22,0.42]	A = 0.81 [0.73;0.88] b = 0.28 [0.19;0.37]	A = 0.77 [0.67;0.87] b = 0.32 [0.24;0.40]	A = 0.76 [0.67;0.86] b = 0.28 [0.19;0.37]
	Sinusoidal	---	---	---	A = 0.68 [0.62;0.74] b = 0.14 [0.10;0.18]	A = 0.65 [0.59;0.71] b = 0.21 [0.16;0.25]	A = 0.62 [0.57;0.67] b = 0.25 [0.20;0.31]
Main experiment	Abrupt	A = 0.91 [0.86;0.95] b = 0.45 [0.40;0.51]	A = 0.91 [0.87;0.95] b = 0.46 [0.40;0.52]	A = 0.89 [0.84;0.93] b = 0.42 [0.35;0.48]	A = 0.93 [0.90;0.96] b = 0.48 [0.42;0.54]	A = 0.91 [0.87;0.95] b = 0.47 [0.41;0.52]	A = 0.91 [0.86;0.95] b = 0.41 [0.36;0.46]
	Sinusoidal	A = 0.75 [0.70;0.81] b = 0.24 [0.17;0.31]	A = 0.72 [0.67;0.78] b = 0.17 [0.11;0.23]	A = 0.74 [0.69;0.80] b = 0.18 [0.12;0.24]	A = 0.73 [0.68;0.78] b = 0.15 [0.10;0.21]	A = 0.73 [0.67;0.78] b = 0.19 [0.13;0.25]	A = 0.84 [0.78;0.89] b = 0.29 [0.23;0.36]

Adaptation parameter values for the pilot and the main experiments, obtained by using a percentile bootstrap with 10,000 repetitions (Efron & Tibshirani, 1993), indicating retention ($A$) and error-correction ($b$) for each block, separated in perturbation schedule and magnitude

### Development of detection performance over trials

**Fig. 4:**
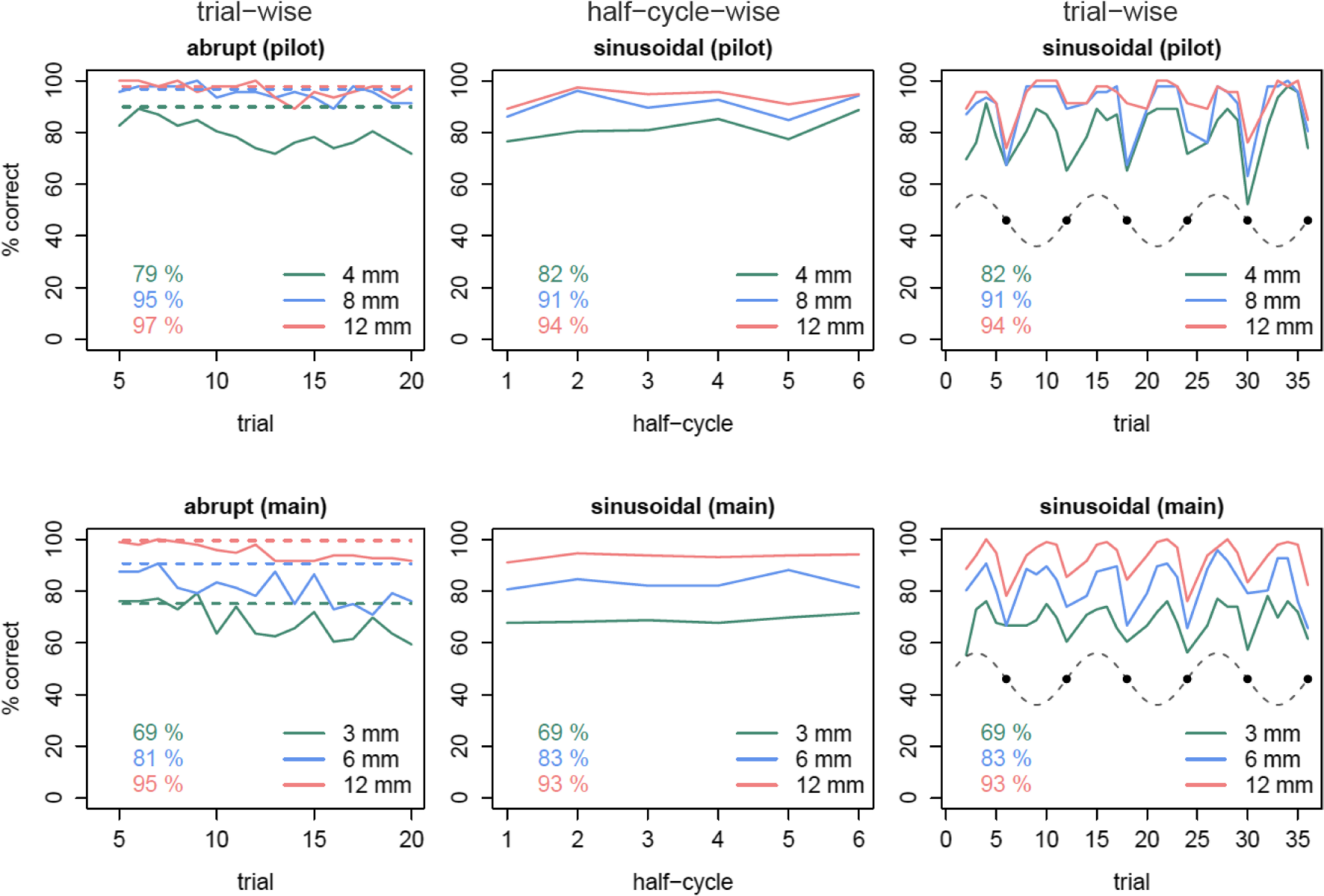
Correct responses over trials. Percentages of correct responses in the two-alternative forced choice (2AFC) task over trials and the overall percentages correct for each perturbation magnitude (green, blue, red). The upper row shows the pilot experiment, the bottom row the main experiment, separated in abrupt and sinusoidal, respectively. On the y axis are the percentages of correct responses for the corresponding trial on the x axis, either for the trial itself (columns 1 and 3, respectively) or related to the absolute mean of all trials in a half sinus-cycle (column 2), that is five positive or five negative mismatches, sinus-scaled related to the corresponding maximum perturbation magnitude of one block. Dashed lines in the abrupt panels indicate the mean percentage correct of sinusoidal trials with the maximum-magnitude mismatch, respectively. These compared with the un-adapted trials of the abrupt schedule (each fifth trial) show roughly similar performance. Over all subsequent trials, the detection performance for the abrupt schedule decreases compared to the fifth trial. Note that a half-cycle (middle column) contains each magnitude in the sinusoidal perturbation schedule exactly once and therefore does not confound perturbation magnitude and detection performance, whereas performance by trial (right column) is confounded by the systematic differences in perturbation magnitude. The gray dashed trace shows the underlying sine wave for perturbation magnitude; note that the percentage of correct responses is modulated at twice the speed of the perturbation sine wave (hence the half-cycle). That is, it closely follows the shape of the *absolute* values of the underlying perturbation, being maximal at the extreme points (independent of whether these were peaks or troughs), and minimal around the zero points (black dots)

We then investigated how the detection performance developed over trials (Fig. 4).

The 2 x 3 rmANOVA on fitted slopes over trials with the factors *perturbation schedule* and *perturbation magnitude* showed a main effect of *perturbation schedule, F*(1,22) = 14.18, *p *= .001 in the pilot experiment, indicative of a stronger decline in correct responses for the abrupt schedule (decreasing 0.5% per trial) than for the sinusoidal schedule (increasing 0.07% per trial). As shown in Fig. 4 (left column), this decline was present both early and later in the experimental blocks. There was no effect on slopes for *perturbation magnitude*, *F*(2,44) = 0.33, *p* = .723, nor an interaction, *F*(2,44) = 2.12, *p* = .133.

In the main experiment, we found the same main effect for *perturbation schedule*, *F*(1,47) = 30.64, *p* < .001, on slopes over trials (decreasing 0.8% per trial for abrupt, increasing 0.03% per trial for sinusoidal) but not for *perturbation magnitude, F*(2,94) = 1.27, *p* = .286, nor for the interaction *F*(2,94) = 1.69, *p* = .191. Note that this main effect refers to the slope of correctness across trials, not absolute percent correct, which obviously differs between magnitudes within each schedule for the pilot (abrupt: *F*(2,44) = 35.58, *p *< .001; sinusoidal: *F*(2,44) = 42.47, *p *< .001) and main experiment (abrupt: *F*(2,94) = 98.66 , *p *< .001; sinusoidal: *F*(2,94) = 221.28, *p *< .001), but cannot be compared between schedules due to different frequencies of perturbation magnitudes (hence the JND analysis below, which takes magnitudes into account).

Comparing the mean percentage of correct responses of maximum-magnitude mismatches in sinusoidal trials (dashed lines Fig. 4) with the non-adapted trials of the abrupt schedule (each fifth trial, the first perturbed trial of each block) for the corresponding magnitude show roughly similar performance (Table 2) in the pilot experiment. This was replicated in the main experiment.

**Table 2:** Mean percentage of correct responses for first-perturbed (abrupt) and maximum-perturbed (sinusoidal) trials with between-participants’ standard deviations

	Pilot		Main	
mismatch	abrupt	sinusoidal	abrupt	sinusoidal
4 mm / 3 mm	82.6 ± 38 %	89.9 ± 30 %	76.0 ± 43 %	75.2 ± 43 %
8 mm / 6 mm	95.7 ± 21 %	96.7 ± 18 %	87.5 ± 33 %	90.6 ± 29 %
12 mm	100 ± 0 %	97.8 ± 15 %	99.0 ± 1 %	99.5 ± 1 %

Mean percentages of correct responses over participants per perturbation magnitude for each first-perturbed trial (fifth trial) in the abrupt schedule and the mean of the maximum-magnitude trials in the sinusoidal schedule with corresponding standard deviation, separately for pilot and main experiments

### Comparing overall detection performance

**Fig. 5:**
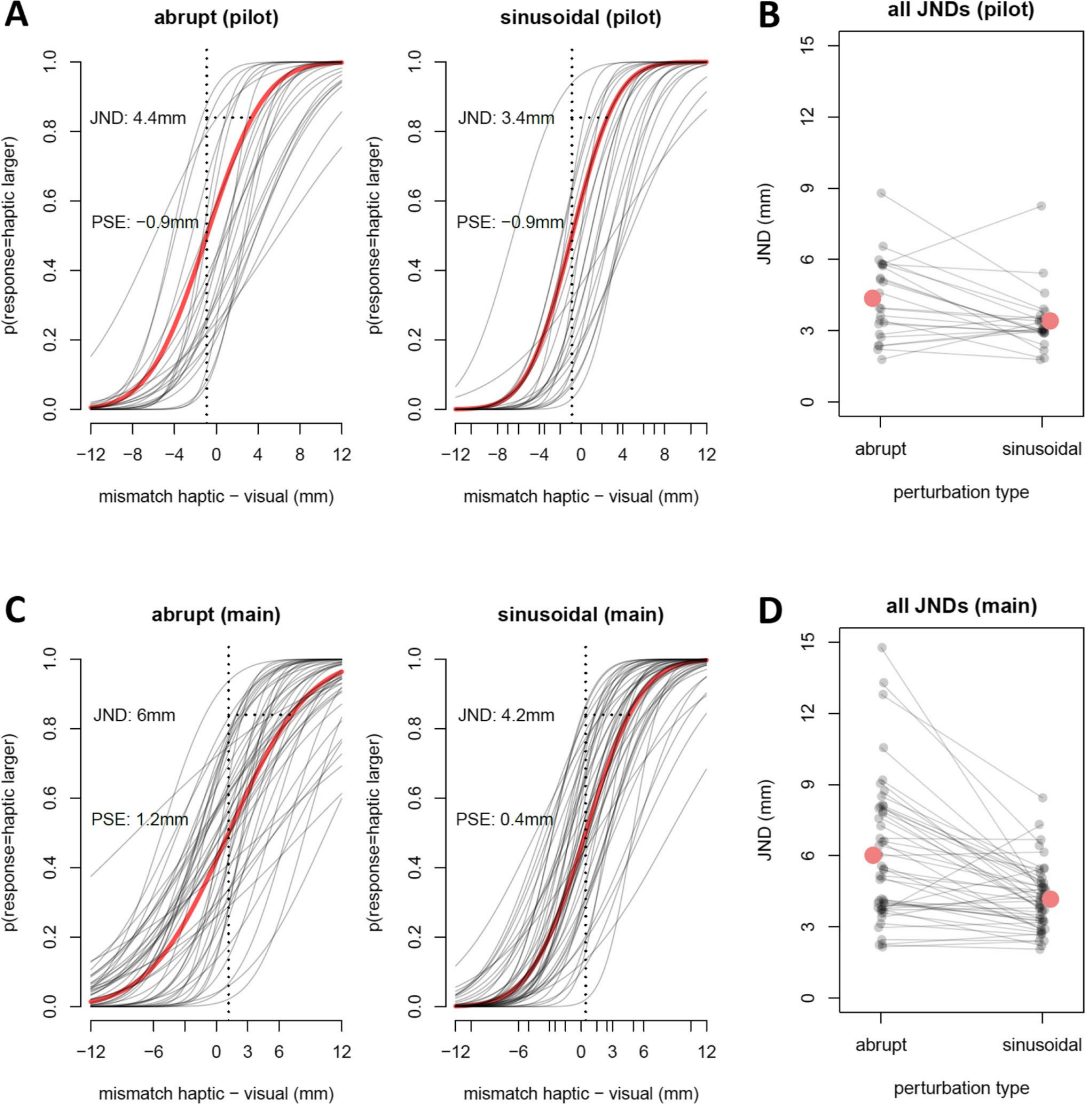
Psychometric function and mean just-noticeable differences (JNDs) for each participant. Analysis of the two-alternative forced choice (2AFC) task separately for pilot experiment (**upper row**) and main experiment (**bottom row**). **A** and **C**: Psychometric functions of each participant for abrupt and sinusoidal perturbations with their corresponding mean JND (horizontal dotted line) and point of subjective equality (PSE, vertical dotted line). The x axis shows the mismatch between the felt and the seen size; the y axis the probability that the felt object was responded to be larger than the seen object. Shaded lines indicate one participant, the red line the overall mean fit. **B** and **D**: Mean JND of each participant for abrupt and sinusoidal perturbations. The dashed dots show one participant, the larger red dot shows the overall mean

To assess overall detection performance, we calculated the JND for each participant per schedule (Fig. 5).

In the pilot experiment, we found a statistically smaller mean JND for sinusoidally introduced perturbations (JND = 3.4 ± 1.3 mm) than for abrupt perturbations (JND = 4.4 ± 1.7 mm; paired t-test for differences: *t*(22) = 2.76, *p* = .011, *d* = .6). A corresponding Bayesian t-test found moderate support for a difference, BF_10_ = 4.4.

In the main experiment, we found the same pattern, with a smaller mean JND for sinusoidal (JND = 4.2 ± 1.4 mm) than for abrupt (JND = 6.0 ± 2.9 mm; paired t-test for differences: *t*(47) = 5.25, *p* < .001, *d* = .8) perturbations. Bayesian analysis showed very strong evidence for an effect of the *perturbation schedule*, BF_10_ > 1000. The results are consistent with a similar baseline level for each schedule (Fig. 4), with the performance decrease over trials for abrupt perturbations resulting in an overall higher JND.

### Relation between perturbation detection and error-correction

Next, we tested whether the relation between adaptation and detection performance also holds at the individual level (i.e., whether individuals with stronger adaptation do worse in the size-comparison task).

Correlations between individuals’ mean slopes of percent correct over trials and the mean error-correction parameter showed in the pilot experiment for the abrupt schedule a correlation of ${r}_{slopes,b}$ = -0.07 and for the sinusoidal schedule of ${r}_{slopes,b}$ = -0.38. In the main experiment, we found a correlation for abrupt of ${r}_{slopes,b}$ = -0.12 and for sinusoidal of ${r}_{slopes,b}$ = -0.01. None of these correlations were statistically significant (all *p* values > .07), with all Bayesian tests (0.4 < *BF*s < 2.4) indicating indecisive evidence.

**Fig. 6:**
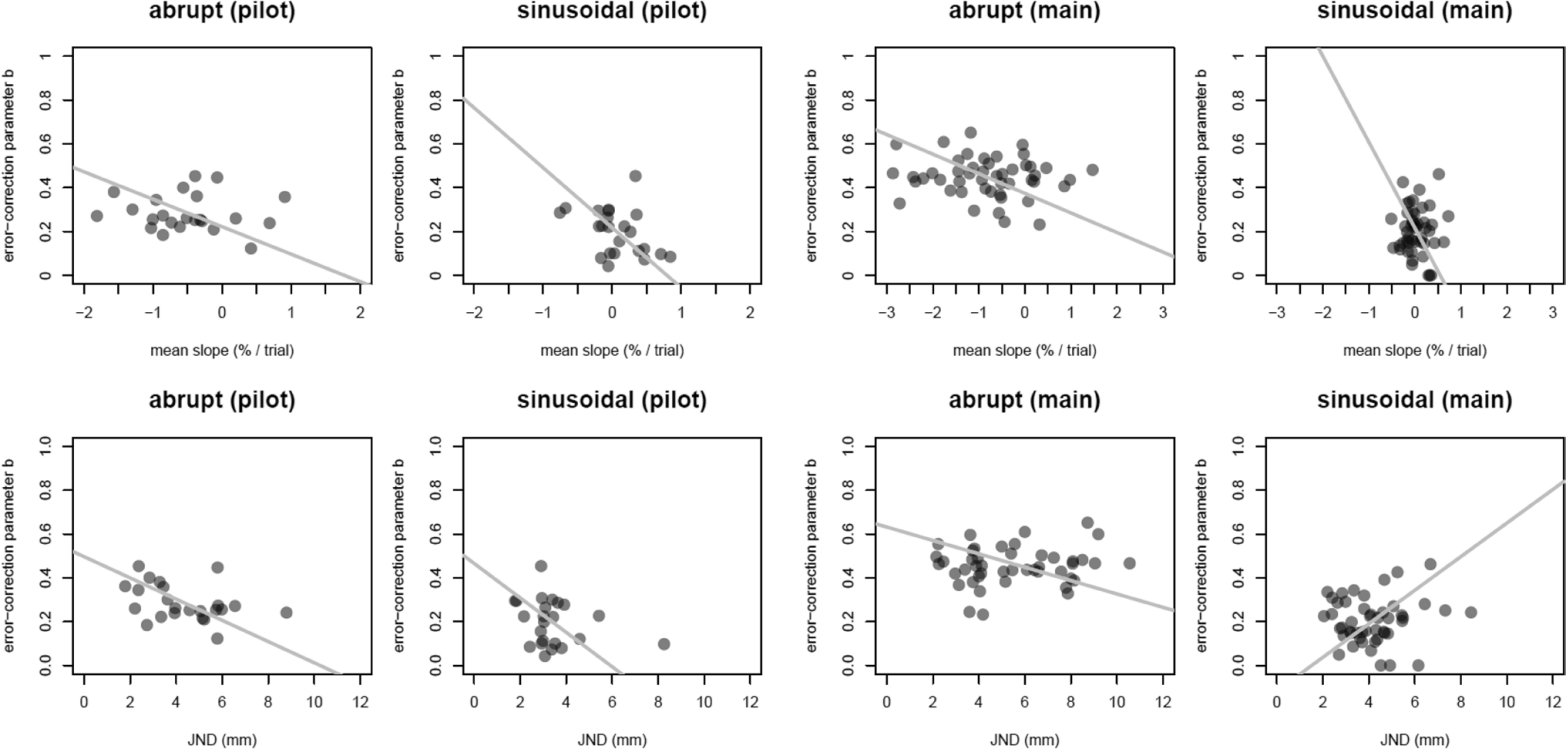
Detection performance and error-correction. Correlation of slopes of percent correct (upper row) or just-noticeable differences (JNDs) (bottom row) with the error-correction parameter ($b$) for the pilot and the main experiment, for each perturbation schedule. The x axis shows the mean slope (%) and JND (mm) and the y axis the error-correction parameter $b$. Each dot represents one participant, the grey line shows the Deming-corrected (Deming, 1943) regression line

The same was true for the correlation between mean JNDs and the mean error-correction parameter $b$ per participant across both schedules (Fig. 6), with no strong relation either in the pilot experiment for abrupt with ${r}_{JND,b}$ = -0.37 or sinusoidal with ${r}_{JND,b}$ = -0.26, nor in the main experiment for abrupt with ${r}_{JND,b}$= -0.12 and sinusoidal with ${r}_{JND,b}$= 0.08. Again, t-tests showed no significant relationships, and Bayesian evidence was indecisive (all *p* values > .075, and 0.35 < *BF*s < 2).

## Discussion

Here, we used different perturbation schedules that allowed different levels of sensorimotor adaptation in order to dissociate the respective effects of a perturbation's *magnitude* and the associated *error signal* on the detection performance of visuo-haptic size mismatches in grasping. Consistent with the idea that the error signal plays a key role, participants’ detection performance was worse overall in schedules when they could adapt their grip to the mismatch more strongly (abrupt) as detectability decreased. Conversely, performance stayed the same over trials when the mismatch changed continuously (sinusoidal), ensuring continuous adaptation. Interestingly and unexpectedly, while participants adapted their grip apertures to both sinusoidal and abrupt perturbations, error-correction parameters were notably higher for abruptly introduced perturbations. Participants’ MGAs also scaled less with object size when a more complex setup was used in the main experiment, perhaps indicative of higher uncertainty about object size particularly on short time scales (Hewitson et al., 2023). Changes in detection performance and strength of adaptation were not correlated across individuals, which could be for several reasons – such as the bidirectional relationship between the two, i.e., stronger adaptation leading to worse detection by minimizing the error signal, but better detection leading to stronger adaptation by enabling explicitly controlled adjustments.

Current models of sensorimotor adaptation incorporate both explicit and implicit components (Miyamoto et al., 2020), which have different properties and complement each other. Concerns about studying one component without the nuisance of the other being present have been discussed for a long time (Held & Gottlieb, 1958; Maresch et al., 2021). The magnitude of the perturbation (Hudson & Landy, 2012), its abrupt or gradual onset (Orban De Xivry et al., 2013), as well as adaptation and thus the associated error signal, (Gaffin-Cahn et al., 2019; Modchalingam et al., 2023) have been suggested to make perturbations detectable and adaptation potentially explicit (Acerbi et al., 2017; Tsay, Avraham, et al., 2021a, 2021b; Tsay, Kim, et al., 2021a, 2021b), but we know of no direct test of these predictions. Here, we show that, indeed, these factors all matter: We see clear effects of perturbation magnitude on detection performance overall, as well as decreasing performance when participants adapt (Fig. 4), and comparable performance in completely un-adapted trials and maximum-magnitude trials of gradually introduced perturbations, respectively. Thus, the intuitive notion that a gradually introduced perturbation could make perturbations harder to detect was not supported by our data. We do, however, show clearly that participants’ ability to judge even initially well-detected perturbations can decrease over repeated exposure. Thus, researchers need to consider participants’ ability both to detect when a perturbation is introduced and to judge whether it remains the same.

Some modeling and experimental design choices should be considered with respect to the generalizability of our results. We modelled error-correction with a difference between observed MGA and MGA predicted from felt object size (i.e., the deviation from a typical, comfortable grasp of the felt object) as the error signal. Using the observed MGA, which inevitably contains noise, implies that participants can use random, non-systematic movement errors to adjust their movements. There is evidence that they do, though it is unclear to what extent (Van Dam & Ernst, 2013). The error signal can also differ between experiments not just in terms of modelling: Conceptually, having feedback once, at the end of the movement, and in a different modality (haptic) than the one used to plan the movement (vision), makes grasping physical objects distinct from certain other actions such as pointing or walking. However, this makes grasping perhaps even more suited to a design with a judgment required after each trial, since alerting participants to a potential perturbation is less of a problem if there is no closed feedback loop. Similarly, in addition to choosing a task, we also had to choose how gradual a “gradually introduced” perturbation really is, which likely affects how well the perturbation at peak magnitudes is masked. We also note that while we argue that changes in detection performance following adaptation are likely consequences of the reduced error signal, another interpretation is that this effect is a form of sensory attenuation (Shergill et al., 2003) caused by the participant’s increasingly precise predictions of the sensory outcome of the grasp. Finally, while unlike many other studies our paradigm allows comparing perturbation detection in earlier versus later trials, the relative length judgments participants gave allow such inferences only on average and not for single trials. Thus, it is also not surprising that a simple state-space model fits our data well, as we cannot say with certainty when exactly participants may have been using explicit strategies to adapt their grip. In future work, it may be useful to model fast and slow processes that have been linked to explicit and implicit adaptation (McDougle et al., 2015) – however, these are known to occur on the order of > 100 trials (Smith et al., 2006) and consequently require more trials per schedule than our design allowed. A design with more trials per block, and potentially perturbation schedules where anticipation of the next trial is impossible for principle reasons, such as a quasi-random perturbation schedule (Acerbi et al., 2017), would allow a more direct test of properties of implicit versus explicit adaptation – here, this was not the main goal. Our key finding of participants struggling to judge perturbations after repeated exposure can also not cleanly be dissociated from participants’ tendencies to alter responses after a while (Bosch et al., 2020): While participant fatigue is not a plausible explanation as sinusoidal schedules (with no signs of performance decline) contained more trials than abrupt schedules, the perturbation and thus the correct answer was the same for 16 straight trials in abrupt-perturbation schedules. To circumvent this issue, other approaches such as using physiological markers like pupillometry as proxies of detection (Yokoi & Weiler, 2022) may be promising.

To understand sensorimotor adaptation, it is becoming increasingly clear that one needs to understand both its implicit and explicit components, as well as their interplay (Miyamoto et al., 2020). Rather than treating cognition and awareness of errors or perturbations as a confounder, a more ecological approach would be to “incorporate the influence of cognitive planning into any realistic and comprehensive model of human sensorimotor learning” (McDougle et al., 2016, p. 542). We concur, and show here that in a common everyday task, one can dissociate the respective effects of a sensory mismatch and the error signal on perturbation detection, with performance markedly deteriorating over repeatedly presented perturbations. This has implications for the design of experimental investigations, as well as understanding the cognitive side of real-world motor behavior.

## Data Availability

Merged data for all experiments are available via the Open Science Framework at https://osf.io/2569y/.

## Footnotes

1 To account for participants adjusting their grip differently when they were aware of the perturbation, we also considered a model that contained two separate parameters for correction after correctly and incorrectly judged trials, respectively. We fitted both models for each block and compared their fit using Akaike’s information criterion (Burnham et al., 2011). We then chose the better-fitting model, which was the simple state-space model (equation 3), for further analyses.

## Code availability

The code for the analysis for all experiments are available via the Open Science Framework at https://osf.io/2569y/.

## Declarations

### Open practices statement

The data and materials for all experiments are available via the Open Science Framework at https://osf.io/2569y/. Neither experiment was preregistered.

## References

[CR1] Acerbi, L., Vijayakumar, S., & Wolpert, D. M. (2017). Target Uncertainty Mediates Sensorimotor Error Correction. *PLOS ONE,**12*(1), e0170466. 10.1371/journal.pone.0170466 28129323 10.1371/journal.pone.0170466 PMC5271325

[CR2] Albert, S. T., & Shadmehr, R. (2016). The Neural Feedback Response to Error As a Teaching Signal for the Motor Learning System. *The Journal of Neuroscience,**36*(17), 4832–4845. 10.1523/JNEUROSCI.0159-16.2016 27122039 10.1523/JNEUROSCI.0159-16.2016 PMC4846676

[CR3] Bingham, G. P., & Mon-Williams, M. A. (2013). The dynamics of sensorimotor calibration in reaching-to-grasp movements. *Journal of Neurophysiology,**110*(12), 2857–2862. 10.1152/jn.00112.2013 24068760 10.1152/jn.00112.2013 PMC3882819

[CR4] Bosch, E., Fritsche, M., Ehinger, B. V., & De Lange, F. P. (2020). Opposite effects of choice history and evidence history resolve a paradox of sequential choice bias. *Journal of Vision,**20*(12), 9. 10.1167/jov.20.12.9 10.1167/jov.20.12.9 PMC7683864 33211062

[CR5] Burge, J., Ernst, M. O., & Banks, M. S. (2008). The statistical determinants of adaptation rate in human reaching. *Journal of Vision,**8*(4), 20. 10.1167/8.4.20 10.1167/8.4.20 PMC2684526 18484859

[CR6] Burnham, K. P., Anderson, D. R., & Huyvaert, K. P. (2011). AIC model selection and multimodel inference in behavioral ecology: Some background, observations, and comparisons. *Behavioral Ecology and Sociobiology,**65*(1), 23–35. 10.1007/s00265-010-1029-6

[CR7] Cohen, J. (1988). *Statistical power analysis for the behavioral sciences* (2nd ed). L. Erlbaum Associates.

[CR8] Deming, W. E. (1943). *Statistical adjustment of data*. John Wiley & Sons.

[CR9] Efron, B., & Tibshirani, R. (1993). *An introduction to the bootstrap*. Chapman & Hall.

[CR10] Ernst, M. O., & Banks, M. S. (2002). Humans integrate visual and haptic information in a statistically optimal fashion. *Nature,**415*(6870), 429–433. 10.1038/415429a 11807554 10.1038/415429a

[CR11] Fechner, G. T. (1860). *Elemente der Psychophysik*. Breitkopf und Härtel. //catalog.hathitrust.org/Record/000662889

[CR12] Gaffin-Cahn, E., Hudson, T. E., & Landy, M. S. (2019). Did I do that? Detecting a perturbation to visual feedback in a reaching task. *Journal of Vision,**19*(1), 5. 10.1167/19.1.5 10.1167/19.1.5 PMC6334820 30640373

[CR13] Hayashi, T., Yokoi, A., Hirashima, M., & Nozaki, D. (2016). Visuomotor Map Determines How Visually Guided Reaching Movements are Corrected Within and Across Trials. *Eneuro*, *3*(3), ENEURO.0032-16.2016. 10.1523/ENEURO.0032-16.2016 10.1523/ENEURO.0032-16.2016 PMC4891765 27275006

[CR14] Held, R., & Gottlieb, N. (1958). Technique for Studying Adaptation to Disarranged Hand-Eye Coordination. *Perceptual and Motor Skills,**8*(3), 83–86. 10.2466/pms.1958.8.3.83

[CR15] Helmholtz, H. (1867). *Handbuch der physiologischen Optik* (Vol. 9). Voss.

[CR16] Hewitson, C. L., Kaplan, D. M., & Crossley, M. J. (2023). Error-independent effect of sensory uncertainty on motor learning when both feedforward and feedback control processes are engaged. *PLOS Computational Biology,**19*(9), e1010526. 10.1371/journal.pcbi.1010526 37683013 10.1371/journal.pcbi.1010526 PMC10522034

[CR17] Hillis, J. M., Ernst, M. O., Banks, M. S., & Landy, M. S. (2002). Combining Sensory Information: Mandatory Fusion Within, but Not Between. *Senses. Science,**298*(5598), 1627–1630. 10.1126/science.1075396 12446912 10.1126/science.1075396

[CR18] Hudson, T. E., & Landy, M. S. (2012). Measuring adaptation with a sinusoidal perturbation function. *Journal of Neuroscience Methods,**208*(1), 48–58. 10.1016/j.jneumeth.2012.04.001 22565135 10.1016/j.jneumeth.2012.04.001 PMC3612424

[CR19] Jeannerod, M. (1986). The formation of finger grip during prehension. A cortically mediated visuomotor pattern. *Behavioural Brain Research*, *19*(2), 99–116. 10.1016/0166-4328(86)90008-2 10.1016/0166-4328(86)90008-2 3964409

[CR20] Kagerer, F. A., Contreras-Vidal, J. L., & Stelmach, G. E. (1997). Adaptation to gradual as compared with sudden visuo-motor distortions. *Experimental Brain Research,**115*(3), 557–561. 10.1007/PL00005727 9262212 10.1007/pl00005727

[CR21] Kopiske, K. K., Cesanek, E., Campagnoli, C., & Domini, F. (2017). Adaptation effects in grasping the Müller-Lyer illusion. *Vision Research,**136*, 21–31. 10.1016/j.visres.2017.05.004 28571701 10.1016/j.visres.2017.05.004

[CR22] Krakauer, J. W., & Mazzoni, P. (2011). Human sensorimotor learning: Adaptation, skill, and beyond. *Sensory and Motor Systems,**21*(4), 636–644. 10.1016/j.conb.2011.06.012 10.1016/j.conb.2011.06.012 21764294

[CR23] Linares, D., & López-Moliner, J. (2016). quickpsy: An R Package to Fit Psychometric Functions for Multiple Groups. *The R Journal*, *8*(1), 122. 10.32614/RJ-2016-008

[CR24] Maresch, J., Werner, S., & Donchin, O. (2021). Methods matter: Your measures of explicit and implicit processes in visuomotor adaptation affect your results. *European Journal of Neuroscience,**53*(2), 504–518. 10.1111/ejn.14945 32844482 10.1111/ejn.14945

[CR25] Mariscal, D. M., Iturralde, P. A., & Torres-Oviedo, G. (2020). Altering attention to split-belt walking increases the generalization of motor memories across walking contexts. *Journal of Neurophysiology,**123*(5), 1838–1848. 10.1152/jn.00509.2019 32233897 10.1152/jn.00509.2019 PMC8086635

[CR26] McDougle, S. D., Bond, K. M., & Taylor, J. A. (2015). Explicit and Implicit Processes Constitute the Fast and Slow Processes of Sensorimotor Learning. *Journal of Neuroscience,**35*(26), 9568–9579. 10.1523/JNEUROSCI.5061-14.2015 26134640 10.1523/JNEUROSCI.5061-14.2015 PMC4571499

[CR27] McDougle, S. D., Ivry, R. B., & Taylor, J. A. (2016). Taking Aim at the Cognitive Side of Learning in Sensorimotor Adaptation Tasks. *Trends in Cognitive Sciences,**20*(7), 535–544. 10.1016/j.tics.2016.05.002 27261056 10.1016/j.tics.2016.05.002 PMC4912867

[CR28] Milgram, P. (1987). A spectacle-mounted liquid-crystal tachistoscope. *Behavior Research Methods, Instruments, & Computers,**19*(5), 449–456. 10.3758/BF03205613

[CR29] Miyamoto, Y. R., Wang, S., & Smith, M. A. (2020). Implicit adaptation compensates for erratic explicit strategy in human motor learning. *Nature Neuroscience,**23*(3), 443–455. 10.1038/s41593-020-0600-3 32112061 10.1038/s41593-020-0600-3

[CR30] Modchalingam, S., Ciccone, M., D’Amario, S., & ’t Hart, B. M., & Henriques, D. Y. P. (2023). Adapting to visuomotor rotations in stepped increments increases implicit motor learning. *Scientific Reports,**13*(1), 5022. 10.1038/s41598-023-32068-8 36977740 10.1038/s41598-023-32068-8 PMC10050328

[CR31] Modchalingam, S., Vachon, C. M., & ‘t Hart, B. M., & Henriques, D. Y. P. (2019). The effects of awareness of the perturbation during motor adaptation on hand localization. *PLOS ONE,**14*(8), e0220884. 10.1371/journal.pone.0220884 31398227 10.1371/journal.pone.0220884 PMC6688819

[CR32] Morey, R. D., & Rouder, J. N. (2018). *Package ‘BayesFactor.’ Retrieved from*. https://cran.r-project.org/web/packages/BayesFactor/index.html. Accessed 2021-07-04

[CR33] Napier, J. R. (1956). The prehensile movements of the human hand. *Journal of Bone and Joint Surgery,**38B*(4), 902–913. 10.1302/0301-620X.38B4.902 13376678

[CR34] Orban De Xivry, J.-J., Ahmadi-Pajouh, M. A., Harran, M. D., Salimpour, Y., & Shadmehr, R. (2013). Changes in corticospinal excitability during reach adaptation in force fields. *Journal of Neurophysiology,**109*(1), 124–136. 10.1152/jn.00785.2012 23034365 10.1152/jn.00785.2012 PMC3545155

[CR35] R Core Team. (2022). *R: A Language and Environment for Statistical Computing* [Computer software]. R Foundation for Statistical Computing. https://www.R-project.org/. Accessed 2022-10-31

[CR36] Rouder, J. N., Speckman, P. L., Sun, D., Morey, R. D., & Iverson, G. (2009). Bayesian t tests for accepting and rejecting the null hypothesis. *Psychonomic Bulletin & Review,**16*(2), 225–237. 10.3758/PBR.16.2.225 19293088 10.3758/PBR.16.2.225

[CR37] Säfström, D., & Edin, B. B. (2004). Task Requirements Influence Sensory Integration During Grasping in Humans. *Learning & Memory,**11*(3), 356–363. 10.1101/lm.71804 15169866 10.1101/lm.71804 PMC419739

[CR38] Savitzky, Abraham, & Golay, M. J. E. (1964). Smoothing and Differentiation of Data by Simplified Least Squares Procedures. *Analytical Chemistry,**36*(8), 1627–1639. 10.1021/ac60214a047

[CR39] Schot, W. D., Brenner, E., & Smeets, J. B. J. (2010). Robust movement segmentation by combining multiple sources of information. *Journal of Neuroscience Methods,**187*(2), 147–155. 10.1016/j.jneumeth.2010.01.004 20096305 10.1016/j.jneumeth.2010.01.004

[CR40] Shadmehr, R., Smith, M. A., & Krakauer, J. W. (2010). Error Correction, Sensory Prediction, and Adaptation in Motor Control. *Annual Review of Neuroscience,**33*(1), 89–108. 10.1146/annurev-neuro-060909-153135 10.1146/annurev-neuro-060909-153135 20367317

[CR41] Shergill, S. S., Bays, P. M., Frith, C. D., & Wolpert, D. M. (2003). Two Eyes for an Eye: The Neuroscience of Force Escalation. *Science,**301*(5630), 187–187. 10.1126/science.1085327 12855800 10.1126/science.1085327

[CR42] Smeets, J. B. J., & Brenner, E. (1999). A New View on Grasping. *Motor Control,**3*(3), 237–271. 10.1123/mcj.3.3.237 10409797 10.1123/mcj.3.3.237

[CR43] Smith, M. A., Ghazizadeh, A., & Shadmehr, R. (2006). Interacting Adaptive Processes with Different Timescales Underlie Short-Term Motor Learning. *PLoS Biology,**4*(6), e179. 10.1371/journal.pbio.0040179 16700627 10.1371/journal.pbio.0040179 PMC1463025

[CR44] Taylor, J. A., & Ivry, R. B. (2011). Flexible Cognitive Strategies during Motor Learning. *PLoS Computational Biology,**7*(3), e1001096. 10.1371/journal.pcbi.1001096 21390266 10.1371/journal.pcbi.1001096 PMC3048379

[CR45] Thoroughman, K. A., & Shadmehr, R. (2000). Learning of action through adaptive combination of motor primitives. *Nature,**407*(6805), 742–747. 10.1038/35037588 11048720 10.1038/35037588 PMC2556237

[CR46] Tsay, J. S., Avraham, G., Kim, H. E., Parvin, D. E., Wang, Z., & Ivry, R. B. (2021). The effect of visual uncertainty on implicit motor adaptation. *Journal of Neurophysiology,**125*(1), 12–22. 10.1152/jn.00493.2020 33236937 10.1152/jn.00493.2020 PMC8087384

[CR47] Tsay, J. S., Kim, H. E., Parvin, D. E., Stover, A. R., & Ivry, R. B. (2021). Individual differences in proprioception predict the extent of implicit sensorimotor adaptation. *Journal of Neurophysiology,**125*(4), 1307–1321. 10.1152/jn.00585.2020 33656948 10.1152/jn.00585.2020 PMC8282225

[CR48] Van Dam, L. C. J., & Ernst, M. O. (2013). Knowing Each Random Error of Our Ways, but Hardly Correcting for It: An Instance of Optimal Performance. *PLoS ONE,**8*(10), e78757. 10.1371/journal.pone.0078757 24205308 10.1371/journal.pone.0078757 PMC3813602

[CR49] Wei, K., Wert, D., & Körding, K. (2010). The Nervous System Uses Nonspecific Motor Learning in Response to Random Perturbations of Varying Nature. *Journal of Neurophysiology,**104*(6), 3053–3063. 10.1152/jn.01025.2009 20861427 10.1152/jn.01025.2009 PMC3007651

[CR50] Wolpert, D., Ghahramani, Z., & Jordan, M. (1995). An internal model for sensorimotor integration. *Science,**269*(5232), 1880–1882. 10.1126/science.7569931 7569931 10.1126/science.7569931

[CR51] Woodworth, R. S. (1899). The accuracy of voluntary movement. *Psychological Review-Monograph Supplements,**3*(3), 1–114.

[CR52] Yokoi, A., & Weiler, J. (2022). Pupil diameter tracked during motor adaptation in humans. *Journal of Neurophysiology,**128*(5), 1224–1243. 10.1152/jn.00021.2022 36197019 10.1152/jn.00021.2022 PMC9722266

[CR53] Ypma, J. (2014). *Introduction to nloptr: An R interface to NLopt*. *R Package, 2*.

